# Dietary Polyphenols—Natural Bioactive Compounds with Potential for Preventing and Treating Some Allergic Conditions

**DOI:** 10.3390/nu15224823

**Published:** 2023-11-17

**Authors:** Anna Dębińska, Barbara Sozańska

**Affiliations:** Department and Clinic of Paediatrics, Allergology and Cardiology, Wrocław Medical University, ul. Chałubińskiego 2a, 50-368 Wrocław, Poland; barbara.sozanska@umw.edu.pl

**Keywords:** polyphenols, asthma, allergic diseases, food allergy, dietary intervention

## Abstract

In light of the constantly increasing prevalence of allergic diseases, changes in dietary patterns have been suggested as a plausible environmental explanation for the development and progression of these diseases. Nowadays, much attention has been paid to the development of dietary interventions using natural substances with anti-allergy activities. In this respect, dietary polyphenols have been studied extensively as one of the most prominent natural bioactive compounds with well-documented anti-inflammatory, antioxidant, and immunomodulatory properties. This review aims to discuss the mechanisms underlying the potential anti-allergic actions of polyphenols related to their ability to reduce protein allergenicity, regulate immune response, and gut microbiome modification; however, these issues need to be elucidated in detail. This paper reviews the current evidence from experimental and clinical studies confirming that various polyphenols such as quercetin, curcumin, resveratrol, catechins, and many others could attenuate allergic inflammation, alleviate the symptoms of food allergy, asthma, and allergic rhinitis, and prevent the development of allergic immune response. Conclusively, dietary polyphenols are endowed with great anti-allergic potential and therefore could be used either for preventive approaches or therapeutic interventions in relation to allergic diseases. Limitations in studying and widespread use of polyphenols as well as future research directions are also discussed.

## 1. Introduction

The prevalence of allergic diseases has increased dramatically over the past two to three decades, and the second wave of the allergy epidemic is now affecting not only the Western world but also developing countries [1,2,3,4,5,6,7,8,9,10,11,12]. Assuming that modifications in genetic predispositions over such a time frame are unlikely, this dramatic upward trend should rather be attributed to changing environmental factors. Dietary patterns and habits seem to be a plausible environmental explanation since they have undergone significant changes in the last decades [13,14,15,16,17,18,19]. Moreover, according to the “diet hypothesis”, nutrients and food components may play a fundamental role in the development of allergic diseases by influencing the immune system and allergic inflammation pathways either directly or through the influence on gut microbiota, and thus may promote or protect against allergic diseases [20,21,22,23,24,25]. Considering that diet is one of the most modifiable and readily accessible interventions, the identification of nutrients, food components, or dietary patterns that could be implemented as a preventive or therapeutic strategy for allergic disease seems to be essential.

Recently, Vlieg-Boerstra et al. proposed an “immune-supportive diet”, a dietary intervention that should be incorporated into the future comprehensive management (prevention or treatment) of allergic diseases. Based on the existing evidence from observational and interventional studies, the authors developed a sustainable diet that should include a highly diverse range of fresh, whole, natural, or minimally processed foods and consist of at least 60% plant-based food such as fresh fruits, raw and cooked vegetables, whole grains, legumes, fermented foods, herbs and spices as well as black and green tea, supplemented with a moderate amount of nuts, peanuts, seeds, omega-3-rich oils, and animal-based products [26]. In addition to foods rich in dietary fiber, fermented foods, and foods naturally rich in beneficial microbes, a prominent place among the recommended foods is given to foods rich in flavonoids as well as polyphenols derived from tea, herbs, and spices, assigned as one of the best anti-inflammatory food components according to the Dietary Inflammatory Index [27]. The safety profile of polyphenols, their widespread distribution in plants, frequent presence in the daily diet, and a broad spectrum of bioactivity, including anti-inflammatory and immunomodulatory properties, make them a valuable and promising dietary intervention in the prevention and treatment of allergic diseases [28,29,30]. Therefore, in recent years, polyphenols have gained great scientific interest and have been subjected to extensive research in response to the growing demand for the development of new preventive and therapeutic alternatives based on natural products [31,32,33].

Purposely, this review aims to summarize the current knowledge and research progress in the field of the potential application of dietary polyphenols as natural, bioactive substances for the prevention and treatment of allergic diseases. We also provide insight into the mechanisms underlying the potential antiallergic properties of phenolic compounds in experimental and clinical settings and the resulting beneficial clinical effects of polyphenols on food allergies and allergic respiratory diseases and offer direction for future research.

## 2. Characterization of Polyphenols

Polyphenols represent an extensive group of at least 10,000 chemical components naturally occurring in the plant kingdom as secondary non-energetic, metabolic products synthetized in response to free radicals or environmental stress factors [34]. In addition to plant defense and protection, phenolic compounds have antimicrobial and antioxidant activity, act as photoreceptors, determine the organoleptic properties, and are responsible for the proper growth and reproduction of plants [35,36]. Polyphenols are abundantly present in almost all plant-based foods; however, their main source in the human diet are fruits, vegetables, seeds, cereals, and nuts, as well as processed foods such as olives, tea, coffee, chocolate, red fermented vinegar, and red wine [34,37,38]. Depending on the structural arrangements that affect absorption, metabolism, bioavailability, and biological activity, polyphenols are divided into four primary classes: flavonoids, phenolic acids, lignans, and stilbenes (Table 1) [35]. Flavonoids are the most common polyphenols, found in over 4000 plants, and responsible for the attractive colors of leaves, flowers, fruits, and vegetables [39]. Mostly studied flavonoids include quercetin, kaempferol, and myricetin, occurring in high amounts in kale, onion, tomato, apples, berries, herbal tea and red wine [40]. Other dietary important flavonoids include isoflavones present in soybeans; anthocyanidins found in colored vegetables and fruits like red cabbage, eggplant, berries, cherries; catechins present in high concentrations in green tea, red wine, and dark chocolate; flavones like apigenin, luteolin, baicalin exist in high numbers in green and black tea, cereals, aromatic herbs such as celery and parsley; naringenin and hesperidin highly present in citrus fruits [38,41,42,43]. Phenolic acids (gallic, caffeic, ferulic acids), constituting almost 30% of total dietary polyphenols, are found in red fruits, onions, and black radishes [34]. Lignans are a small class of phenolic compounds mainly present in linseed, whole grains, and cereals [38]. Among stilbenes, the most important for human health is resveratrol, the main source of which are grape skins, red wine, peanuts, blueberries, and cranberries [44]. Polyphenols have been known since the 1930s when the new element extracted from oranges was classified as a flavonoid; however, only extensive research over the last two decades has provided data on the beneficial effects of the phenolic compound on human health, confirming their antibacterial and antifungal, anti-inflammatory, antioxidant and immunomodulatory properties, as well as antidiabetic, anticarcinogenic, anticoagulant, and neuroprotective functions [28,29].

## 3. Evidence from Epidemiological Studies

Evidence from several epidemiological studies investigating the relationship between nutrition and allergic diseases has suggested that increased consumption of fruit and vegetables is associated with a lower prevalence of food allergy, allergic rhinitis and asthma [51,52,53,54,55,56,57]. A large observational study conducted in children demonstrated that higher consumption of fruit was associated with reduced risk of allergic rhinitis, atopic dermatitis, and recurrent wheeze. The protective effect was observed in children who consumed fruit at least three times per week as a part of the more traditional diet, while fast food/burger eating significantly increased the rate of wheezing and allergic rhinitis [58]. Interestingly, the dietary-related reduction in the risk of developing allergic diseases was particularly pronounced for a diet containing fruit such as apples, pears, carrots, tomatoes, and citrus [54,59,60]. Three population-based case–control studies in Australia, Finland, and the United Kingdom have shown that apple and pear intake was correlated with a decreased risk of asthma and significantly lower frequency and severity of asthma symptoms and bronchial hypersensitivity [21,60,61,62]. Moreover, Willers et al. identified the consumption of apples during pregnancy as a protective factor for the development of childhood asthma and allergic diseases [63]. Recently published systemic review provided an excellent overview of studies that investigated the nutritional interventions in asthma patients showing the most consistent and promising results for certain components from herbs, herbal mixtures, and extracts [64]. These beneficial, allergy-preventing effects associated with a diet rich in fruits, vegetables, and herbs have been attributed to the high content of polyphenols, in particular, flavonoids in this dietary source [56,59,64,65].

The Mediterranean diet, characterized by high consumption of vegetables, cereals, and olive oil, has also being widely investigated as a dietary pattern that may potentially exert a beneficial impact on the pathogenesis of asthma and other allergic diseases. Recent systematic reviews and meta-analysis have provided highly promising evidence suggesting that adherence to the Mediterranean diet is inversely associated with the prevalence of asthma, atopy, and food allergies [52,66,67]. Moreover, data from observational and experimental studies have pointed out that olive oil, rich in polyphenols and fatty acids, as a main component of the Mediterranean diet, may be responsible for the health benefits of this dietary pattern, including the remarkable effectiveness against the development of asthma and other allergies [68]. For instance, a recent population-based multi-case-control study confirmed the correlation between olive oil consumption and reduced risk of current asthma, indicating that each additional 10 g per day of olive oil intake reduces the risk of asthma by a further 20% [69].

Interesting results regarding the correlation between prenatal dietary exposure to different food components, mainly chemicals, and the risk of allergic diseases came from a very recently published, large cohort study involving 1248 mother–child pairs observed up to 8 years of age. Prenatal dietary exposure to resveratrol was associated with a lower risk of both wheezing and allergic rhinitis, while most tested food chemicals increased the risk of asthma, wheezing, and allergic rhinitis [70].

## 4. Potential Mechanisms of Action in the Prevention or Treatment of Allergic Diseases

**Figure 1:**
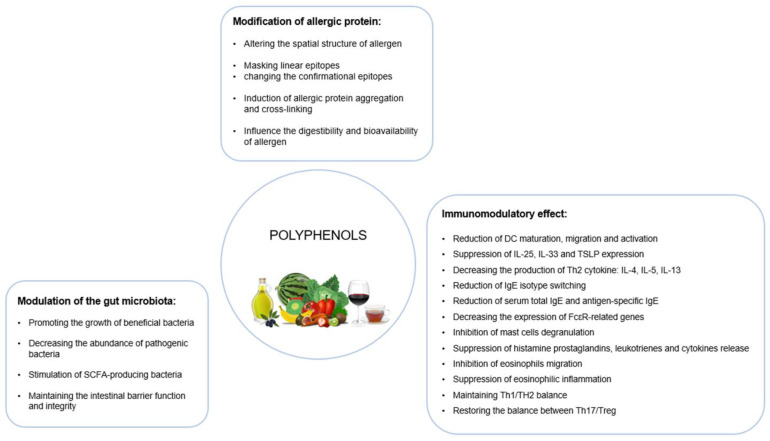
Proposed mechanisms of anti-allergic action of polyphenols.

The exact molecular and cellular mechanisms by which polyphenols may exert their protective and therapeutic effects on allergic diseases remain uncertain and need to be elucidated. However, it is suggested that the beneficial antiallergic activity of polyphenols is related to their influence on three fundamental targets: (1) interaction with allergic proteins and reduction in their allergenicity, (2) modulation of the local and systemic immune response, and (3) impact on the gut microbiota composition and diversity (Figure 1).

### 4.1. Modification of Allergic Protein

The potential of dietary polyphenols to reduce food allergenicity is related to their strong affinity for binding food proteins and the ability to form soluble and insoluble protein–phenolic complexes with changed functional properties [71,72]. It has been proposed that the conjunction of polyphenols with allergens present in food, which can be both covalent and non-covalent, causes changes in the spatial structure of the allergenic protein, thereby reducing the IgE-binding capacity to allergen that reflects its sensitization potential [72,73]. Indeed, different polyphenols have been found to mask linear epitopes of the allergen by conjugation with nucleophilic amino acids as well as change the conformational epitopes of allergen by altering the secondary and tertiary structure of the protein, hence lowering allergenicity [72,74,75,76]. Several studies focusing on the phytochemical modification of β-lactoglobulin, the major allergen in cow milk, have demonstrated that covalent conjugation with various polyphenols, namely rutin, ferulic acid, caffeic acid, epigallocatechin (EGCG), chlorogenic acid lead to conformational changes resulting in more unfolded structure of proteins that correlate with reduced IgG/IgE binding capacity [77,78,79,80,81,82]. Furthermore, Pu et al. confirmed that several flavonoids, such as EGCG, naringenin, myricetin, kaempferol, and quercetin, can also decrease the allergenicity of β-lactoglobulin by noncovalent interactions, showing the highest inhibitory potency on β-LG antigenicity for EGCG resulting in 73% reduction in IgE binding ability [83]. Similarly, covalent conjugation between quercetin and ovalbumin changed the protein’s secondary and tertiary conformation and caused the less folded structure and decreased allergen stability, which declines the ovalbumin allergenicity tested in vitro as the ability to trigger degranulation of effector cells and in vivo as the degree of the allergic immune response and symptom score [84]. Moreover, the spectrometric structural analysis of profilin family allergens after covalent and non-covalent binding with quercetin indicated the loss of the α-helical structures in the conjugates by up to 40%, which, together with masking of the antigenic epitopes, resulted in markedly lower allergenicity [85]. Recently, it has been reported that conformational changes in the structure of shrimp tropomyosin caused by covalent interaction with CA, EGCG, and polyphenols extracted from the algae Sargassum fusiforme can lead to a significant reduction in allergenicity, which, in turn, alleviated shrimp-induced allergic symptoms in vivo [86,87].

Besides the ability for structural modification, polyphenols can also improve the overall functional properties of allergic proteins determining the allergenic potency, in particular digestibility, which can be increased as a result of exposing a larger number of protein cleavage sites, and thus faster and more effective degradation of allergen [71,88,89]. For instance, the results from experimental studies have indicated that the covalent conjugation of EGCG and CA to peanut proteins significantly decreases allergenicity not only by changing both linear and conformational epitopes but also by improving the digestibility of peanut allergen. The reduced peanut protein allergenicity as expressed by lower food allergy responses including symptoms, frequency of mast cells, and damage in the intestine was observed in vitro and in the food allergy mouse model [90,91,92]. Similar results, confirming the effect of polyphenols on the simulated gastric digestion and the spatial structure of Ara h 1 peanut protein, were obtained for five major apple polyphenols (epicatechin, phlorizin, rutin, chlorogenic acid, and catechin), indicating epicatechin as exerting the strongest inhibitory effect on peanut allergy [93]. Additionally, the covalent binding of wheat gliadin with chlorogenic acid and luteolin influenced the IgE/IgG binding capacity by changing the protein conformation and transforming it into a more ordered structure, as well as significantly improving the thermal stability and in vitro digestibility of allergic proteins [94,95].

Finally, the binding of polyphenols to the allergen may induce protein aggregation and cross-linking, leading to a reduction in allergen load, possibly through the loss of some reactive allergens and a reduction in the accessibility of reactive epitopes [71,74,75]. On the other hand, the ability of polyphenols to form cross-linked protein polymers causes the allergen binding to be effective, even if the number of polyphenol molecules is less than the number of allergen reaction sites, and the resulting polyphenol–allergen complexes are more stable and consequently more effective [71,75,96]. This phenomenon was well illustrated in a great series of studies evaluating the structural and functional properties of various soybean globulins after covalent binding with polyphenols such as EGCG, chlorogenic acid, caffeic acid, gallic acid, and tannic acid [96,97,98,99]. In all cases, the formation of polyphenol–soybean globulin conjugate and cross-linking of soybean proteins resulted in structural changes hiding or destroying allergen epitopes as well as increased UV absorption and protein digestibility, which, in turn, reduced IgE binding activity and histamine release in vitro [96,97,98,99]. Interestingly, experiments on the murine model of allergy revealed that covalent conjugation of soy 11S protein with EGCG and chlorogenic acid can not only reduce the allergenicity of the protein and alleviate the allergy symptoms, but also effectively induce the development of oral tolerance to soy allergen [99].

Considering all this evidence, dietary polyphenols have great potential to reduce food allergenicity; therefore, it could be useful in developing hypoallergenic foods that could potentially alleviate food allergy symptoms and/or prevent its development by inducing tolerance.

### 4.2. Immunomodulatory Effects

In recent years, much attention has been paid to the mechanisms by which polyphenols can exert immunomodulatory actions in allergic diseases. Evidence from in vitro and in vivo studies has highlighted that polyphenols can influence allergic immune response, exhibiting both stimulatory and inhibitory effects at two essential stages, during the sensitization and the effector phase [30,31,32,33,100,101,102,103,104,105,106,107].

#### 4.2.1. Sensitization Phase

The first stage in the sensitization phase is the presentation of the entering allergen by dendritic cells (DCs) to naïve CD4+ T cells in draining lymph nodes, which leads to the differentiation of naïve CD4+ T cells into allergen-specific Th2 cells producing proallergic cytokines (IL-4, IL-5, IL-9, IL-13) [108]. It has been demonstrated that specific groups of polyphenols can impede the antigen presentation process by affecting DC differentiation, maturation, and capacity to activate T cell differentiation into allergic type Th2 cells [103]. Indeed, resveratrol impacts the differentiation of human DC from monocytes, as well as inhibits the DC maturation leading to the induction of an immature phenotype [109,110]. The ability to suppress the phenotypic and functional maturation of murine bone marrow-derived DC has been demonstrated for different polyphenols, such as quercetin, curcumin, fisetin, silibinin, isoflavones, and blackberry polyphenols. In addition, these compounds hinder efficient antigen presentation by downregulating the expression of co-stimulatory molecules (CD83, CD80, CD86) and major histocompatibility complex (MHC) class II on the surface of DCs [111,112,113,114,115]. Other polyphenols, EGCG, and apigenin were found to not only affect the DCs differentiation and decrease antigen uptake activity, but also provoke apoptosis of DC-precursors and immature DCs [112,116]. Furthermore, polyphenols can exhibit the regulatory effect on naïve CD4+ T cell priming, the next important event in the sensitization phase. In fact, it has been shown that kaempferol and lycoricidine inhibit the naïve CD4+ T cells activation and differentiation into Th2 effector cells by suppressing TCR-mediated signaling cascades [117,118].

In addition to allergen presentation by DCs, cytokines such as TSLP, IL-25, and IL-33, secreted in response to food- and aero-allergens by epithelial cells lining barrier sites, play an important role in the allergic sensitization phase by activating DCs and innate lymphoid cells type 2 (ILC2) and promoting Th2 cell development [119,120]. ILC2 are also highly essential in the promotion of the Th2 immune response by producing IL-4, IL-13, and IL-5 in the early stage of antigen sensitization [121]. Various polyphenols such as quercetin, curcumin, and baicalin have been identified as suppressing the expression and secretion of TSLP and IL-33 both in atopic dermatitis (AD) models of human keratinocytes and AD-like mouse models [122,123,124]. Moon et al. demonstrated that two other polyphenols, resveratrol and naringenin, inhibit the TSLP production and mRNA expression in human mast cell lines [125,126]. The modulatory effect of quercetin on epithelium-derived cytokines was also observed in experimental models of allergic airway inflammation as quercetin significantly decreased IL-25, IL-33, and TSLP levels in BAL and expression of this cytokine in lung tissue [127]. Recently, Fallopia japonica (Asian knotweed), a traditional medicinal herb rich in polyphenols such as resveratrol and flavones, was reported to target the IL-33/TSLP signaling pathway, strongly reducing these cytokine levels in both nasal and bronchoalveolar lavage fluid in the allergic rhinitis and asthma mouse model [128].

The proallergic cytokines, IL-4 and IL-13, produced by Th2 and ILC2 in the early stage of the sensitization phase, prompt IgE isotype class-switching in B cells and their transformation into plasma cells secreting a huge amount of allergen-specific IgE that subsequently link to high-affinity FcεRI receptors on the surface of mast cells and basophils, which leads to an allergic sensitization state [129]. Polyphenols have been suggested to affect B cell recruitment, maturation, and function; however, this effect has not yet been thoroughly investigated and described [71,100,130]. On the other hand, the capacity to inhibit the production of antigen-specific IgE in a dose- and time-dependent manner has been well documented in in vitro and in vivo studies for a number of polyphenols such as curcumin, rosmarinic acid, quercetin, ferulic acid, tea catechins (EGCG, ellagitannins and gallic acid) and red grape polyphenols [104,131,132,133,134,135,136]. Zhang et al. illustrated the modulatory effect of polyphenols with an example of dihydromyricetin, a natural flavonoid, which effectively suppressed the sensitization phase by reducing the population of B cells and their production of antigen-specific IgE as well as blocking the FcεRI–IgE interaction [137]. Similarly, phlorotannins (i.e., eckol, dieckol) and tea catechins could interact with FcεRI by directly binding to the α chain, thereby blocking the possibility of binding antigen-specific IgE to FcεRI and thus suppressing the sensitization phase of mast cells [138,139,140]. Additionally, evidence has been provided that phlorotannins, saponins, catechins, as well as quercetin, kaempferol and resveratrol may contribute to the attenuation of the allergic reaction by reducing the expression of the FcεRI receptor, which is crucial for the persistent sensitization of MCs and their subsequent degranulation during the effector phase [105,140,141,142,143].

#### 4.2.2. Effector Phase

During the effector phase, re-exposure to the same allergen leads to the cross-linking of IgE bound to FcεRI on the surface of mast cells and basophils causing their activation and degranulation with the release of reactive mediators triggering acute systemic allergic reaction [119]. Recently, numerous in vitro and in vivo studies have investigated the mechanisms through which polyphenols may exert a modulatory effect on mast cells as major effector cells of the allergic reaction [30,31,105]. In addition to aforementioned impact on the expression of the FcεRI receptor and the FcεRI–IgE binding, different polyphenols, such as resveratrol, quercetin, procyanidins from cinnamon or apple extract, can suppress mast cells activation via inhibiting the cross-linking of IgE by allergens on the cell surface [106,144,145]. Moreover, the ability to stabilize mast cell membranes and thus suppress their degranulation has been demonstrated for certain polyphenols, including quercetin, phlorotannins, luteolin, and myricetin, which have been found to downregulate the expression of calcium channel proteins and inhibit calcium influx and intracellular calcium elevation necessary for the degranulation in mast cells [107,138,146,147,148]. Indeed, these phenolic compounds, along with curcumin, EGCG, rosmarinic acid, and resveratrol, exhibited pronounced inhibitory effects on the release of histamine and β-hexosaminidase, which are used as markers to evaluate the level of mast cell degranulation [104,107,141,144,146,147,148,149,150,151]. In addition, polyphenols have been reported as potent suppressor of both FcεRI mediated protein kinases (Syk, Lyn, PLCγ, PKC) signaling cascade and the MAPK and the NF-*κ*B signaling pathway that are critical for the allergic reaction, resulting in attenuating secretion of pro-inflammatory cytokines (IL-4, TNF-α) in and synthesis of lipid mediators (prostaglandin D2, leukotrienes) [144,146,149,150,152,153,154]. Interestingly, Yong et al. confirmed the anti-allergic potential of stingless bee honey (Kelulut honey) in terms of mast cell activation and degranulation; however, the inhibitory effect was strictly dependent on the botanical source of honey as it was only indicated in the case of rich in polyphenols honey obtained from bamboo and rubber trees, while honey poorer in polyphenols sourced from noni and mango did not show such anti-allergic action [155].

Within the later effector phase, the overexpression of Th2-related immune response, accompanied by increased production of Th2 cytokines, i.e., IL-4, IL-5, and IL-13, results in maintaining high antigen-specific IgE levels, recruitment of immune cells such as eosinophils to inflammatory sites, increased mucus production, and initiates chronic allergic inflammation causing tissue damage and remodeling [156]. A large number of experimental studies using cellular and animal models have confirmed that polyphenols exhibit immunomodulatory effects at various crucial stages of the effector phase, including inhibition of the Th2 differentiation, downregulation of the Th2-related cytokine production, reduction in the inflammatory cells infiltration, and, as a result, the suppression of allergic inflammation. Most importantly, polyphenols were found to effectively restore the Th1/Th2 imbalance through upregulating the Th1 pathways while hampering the overexpression of Th2-mediated immune responses [30,100,130]. These effects have been well documented for curcumin in several models of allergic diseases indicating its anti-allergic action exerted by reducing the activity and proliferation of Th2 cells along with decreasing the secretion of IL-4, IL-5 and IL-13, inhibiting the activation and infiltration of macrophages, monocytes, neutrophils and eosinophils into inflammatory sites and shifting the Th1/Th2 response towards the Th1 phenotype [124,132,157,158]. With particular emphasis on asthma models, kaempferol and rosmarinic acid attenuated airway inflammation by lowering the synthesis of IL-4, Il-5, and Il-13 in the serum and bronchoalveolar lavage fluid (BALF) and effectively reduced the recruitment of eosinophils into lung tissues, airway hyperresponsiveness and hyperproduction of mucus [136,159,160]. Evidence from studies in a mouse model of allergic rhinitis indicated that flavonoids such as quercetin, isoquercetin, myricetin, and luteolin alleviate nasal mucosa inflammation not only by suppressing Th2 cell differentiation and cytokine secretion but also by promoting the Th1 pathway and thus maintaining Th1/TH2 balance [161,162,163,164,165]. Moreover, quercetin as well as tea catechins (ellagitannins and gallic acid) have been shown as effective inhibitors of ovalbumin (OVA)-induced allergic response, stimulating immune tolerance through the Th1/Th2 modulation and induction of regulatory T-cells (Treg) in a mouse models of food allergy [133,162].

Indeed, in addition to Th1/Th2 dysregulation, disturbance of the balance between Th17/Treg cells contributes to the breakdown of immune tolerance and thus has a role in the enhancement and progression of chronic allergic inflammation [166,167]. Recent experimental studies provided strong evidence that certain flavonoids such as quercetin, luteolin, cyanidin, and baicalin exert an anti-allergic effect by upregulating the number of Tregs and restoring the balance between Th17/Treg [164,168,169,170]. Similarly, curcumin showed a modulatory effect on Th17/Treg imbalance, effectively reducing the differentiation of Th17 cells while significantly increasing the number of Treg subtypes in a murine model of asthma [171,172,173].

In conclusion, the abundance of data from in vitro experiments and studies in animal models indicate that polyphenols possess the potential to prevent the development of allergic diseases by modulating the allergic sensitization process, and their impact on allergy effector cells during re-exposure may constitute a new therapeutic strategy.

### 4.3. Modulation of the Gut Microflora

Modulation of the gut microbiota represents another target mechanism by which polyphenols may exert antiallergic effects and thus play a role in the prevention or treatment of allergic diseases. The gut microbiome is intrinsically related to the maturation and regulation of the immune response; thus, any disturbance of the gut-immune axis resulting from dysbiosis, defined as alterations in the composition and diversity of the gut microbiota, has been suggested to increase the risk of developing allergic diseases [174,175,176,177,178]. Recently, the large, deeply characterized CHILD cohort study provided evidence that delayed and insufficient infant microbiota maturation is strongly associated with an increased risk of developing asthma, allergic rhinitis, food allergy, and atopic dermatitis at the age of 5 years and, importantly, an immature gut microbial composition preceded the diagnosis of an allergic disease [179]. Regarding this evidence, it is plausible that dietary interventions affecting the microbiome composition and function can be considered as an indirect method of preventing allergic diseases. This may be particularly true for polyphenols, as a major part of polyphenols passes through the small intestine unchanged and is only metabolized and absorbed after reaching the large intestine, which may explain the certain ability of polyphenols to modulate the gut microbiota [180]. In fact, polyphenols could act as “prebiotics” to shape the composition of gut microbiota through promoting the growth of beneficial genera including *Bifidobacterium*, *Lactobacillus*, and *Akkermansia* while inhibiting the frequency of various pathogens and altering the ratio of Firmicutes to Bacteroides [181,182]. To name only a few of many examples, curcumin and resveratrol restore intestinal dysbiosis by decreasing the relative abundance of Actinobacteria, Proteobacteria, and Firmicutes/Bacteroidetes ratio and exhibit anti-inflammatory effects in animal experiments [183,184,185]. Rutin and quercetin stimulated the populations of *Bifidobacterium*, *Bacteroides*, and *Lactobacillus* and strongly inhibited *Enterococcus* and *Fusobacterium* ssp.; thus, they promote gut homeostasis [186,187]. Other flavonoids such as procyanidin, green tea catechins, and grape seed extract can also decrease the Firmicutes/Bacteroidetes ratio and exhibit growth enhancement of *Akkermansia*, *Lactobacillus plantarum*, and *Lactobacillus casei* to alleviate inflammation [188,189,190,191,192]. Similarly, gallotannins derived from mango peels and cocoa-derived polyphenols were found to demonstrate prebiotic effect on the populations of bifidobacteria and lactic acid bacteria [193,194,195].

The mechanisms by which this beneficial effect on gut microbiota influences local and systemic immunity and promotes immune tolerance are now being studied. It has been demonstrated that gut microbiota can significantly impact the maturation of the immune system and can regulate the immune response by promoting the differentiation of Tregs, decreasing the amount of circulating basophil and other allergy effector cells along with stimulation of anti-inflammatory cytokine (IL-10, TGF-β) production [180,196,197,198,199]. Furthermore, the gut microbiota is effective in maintaining the intestinal barrier function and integrity and can increase mucin secretion, level of fecal IgA, and production of antimicrobial peptides, and thus can prevent allergen access to the systemic circulation [178,196,197,198]. Anthocyanins, for example, were found to be effective in the regulation of gut microbiota composition by promoting the growth of beneficial bacteria (*Lactobacillus* and *Odoribacter*) and lowering the abundance of pathogenic bacteria which was consequently associated with the upregulation of intestinal barrier-related gene expression, increased secretion on IgA and β-defensin and restoring Th1/Th2 imbalance [200,201]. Another in vivo study stated that the supplementation of baicalin to rats stimulated the population of SCFA-producing species such as *Butyricimonas* spp., *Roseburia* spp., and *Eubacterium* spp. that in turn promote intestinal immune tolerance by the downregulation of the Th17/Treg ratio and strengthen the intestinal barrier by the upregulation of TJ protein expression [202]. Furthermore, dietary supplementation with ferulic acid may counteract the impairment of the intestinal barrier and changes in gut microbiota observed after the LPS challenge in piglets [203]. Additionally, cocoa-derived polyphenols were demonstrated to induce oral immune tolerance in OVA-sensitized mice by changing gut microbiota composition, and this tolerogenic effect could be due to a reduction in the percentage of bacteria belonging to the Firmicutes and Proteobacteria phyla and an increase in the population of Tenericutes and Cyanobacteria [204].

An increasing body of experimental and epidemiological data has suggested the existence of a cross-talk phenomenon known as the gut–lung axis, which may, at least partially, explain the complex pathogenetic relationship between the gut microbiota and lung immunity and its impact on the development of lung diseases such as asthma [205,206]. Although the exact mechanisms need to be elucidated, one of the proposed pathways includes the systemic transmission of gut microbiota-derived products and metabolites that act as signaling molecules transferring intestinal microbial signals to the lung and regulating immune lung homeostasis [205,207,208,209]. In particular, SCFAs have been suggested as important immunomodulatory metabolites that function as a bridge between the microbiota and the immune system, exhibiting a potential antiallergic effect by modulating the epithelial barrier function and immune tolerance in the intestines, as well as both innate and adaptive immunity that is involved in the development of asthma [198,210,211]. Importantly, polyphenols not only modify gut microbial composition, but also exhibit beneficial effects on SCFA production and increase the level of circulating SCFAs [180,181]. For instance, anthocyanins, phenolic acids, green and black tea catechins, grapefruit extract, containing hesperidin and naringin, and apple polyphenols were proven to expand the abundance of SCFAs-producing bacteria such as *Akkermansia* and increased the production of SCFAs, especially acetate, propionate, and butyrate [181,212,213,214,215,216]. More recently, Alharris et al. confirmed in a murine model of allergic asthma that resveratrol can ameliorate asthma features, as expressed by improvement in pulmonary functions and reduction in inflammatory cytokines in the lungs. In addition, significant upregulation of tight junction proteins and reduction in mucin production was observed in the pulmonary epithelium which was directly related to the stimulating effect of resveratrol on the growth of *Akkermansia mucinuphila* in the lungs. A detailed analysis of gut microbiota has also revealed that resveratrol causes *Bacteroides acidifaciens* outgrowth accompanied by an increased production of SCFAs, mainly butyric acids inducing the Tregs cells subtype which in turn can modulate the adaptive immune response and attenuate asthma. The authors suggested that the therapeutic effect of resveratrol on allergic asthma can be attributed to the alteration in both lung and gut microbiome, highlighting the role of complex cross-talk between gut microbiota and lung immunity [217].

Regarding this evidence, dietary polyphenols and their metabolites may modulate the composition of gut microflora and intestinal barrier function, and consequently also the local and systemic immune response; however, the potential implication of these mechanisms in the prevention and treatment of allergic diseases in humans needs to be resolved.

## 5. Polyphenols in the Prevention and Treatment of Food Allergy

Based on the evidence from experimental studies confirming the ability of polyphenols to reduce food allergenicity, modulate systemic immune response, and modify the gut microbiota, polyphenols seem to be dietary components potentially useful for the prevention and treatment of food allergy. Besides the previously mentioned anti-allergic and anti-inflammatory mechanisms, polyphenols can additionally modulate the local intestinal immune response, repair gastrointestinal mucosa, maintain its integrity, and improve intestinal barrier function [188,189].

Numerous studies using animal models of food allergy have considered the impact of various polyphenols on immune response which dictate the sequelae of allergic reaction showing their potential to ameliorate food hypersensitivity and allergy symptoms in sensitized mice [31]. In this respect, Zhang et al. indicated that tea catechins such as epigallocatechin (EGC) and epigallocatechin gallate (EGCG) are effective in inhibiting mast cell activation, specific IgE and Th2 cytokine production and reducing the degree of pathological changes in the intestine in a model of mice sensitized by αs1-casein milk protein [218]. Similarly, Chinese sweet tea polyphenols, particularly ellagitannins and gallic acid, have been demonstrated as a potent inhibitor of hen egg ovalbumin-induced allergic response in mice by modulating the Th1/Th2 balance, increasing the percentage of Treg subtype and enhancing intestinal IgA secretions which clinically manifested as a reduction in symptoms such as scratching, lethargy and gastrointestinal signs [133]. The remarkable therapeutic effect against food allergy has also been reported for other polyphenols such as resveratrol, myricetin, quercetin, curcumin, and polyphenols extracted from apple and areca nuts that were found to not only mitigate food allergy symptoms including diarrhea, decreased rectal temperature and anaphylactic reaction, but also suppress allergic response by inhibiting the infiltration and degranulation of mast cells in the duodenum, decreasing the serum level of specific IgE, restoring the Th1/Th2 imbalance and upregulating the population of Treg [162,219,220,221,222]. Interestingly, a study in a rat model of food allergy indicated that cocoa-derived flavonoids administered simultaneously with an allergen during the induction phase can completely prevent the synthesis of specific IgE as well as inhibit local and systemic immune response as evidenced by the suppression of Th2-related cytokines released from a mesenteric lymph node and spleen cells, thus exhibiting a protective effect against food allergy, although this impact was not sufficient to prevent anaphylactic reaction after an oral allergen challenge [223].

In addition, several studies have presented evidence for the ability of polyphenols to modulate local immune response by suppressing intestinal Th2-mediated immunity, affecting TCR-mediated signaling cascades and inducing the differentiation and functionality of Treg cells in lamina propria, resulting in the maintenance of immune tolerance which is closely related to oral tolerance formation and the alleviation of food allergy [182,224,225,226,227]. Namely, it has been demonstrated that a diet enriched with polyphenols such as cocoa flavonoids or apple condensed tannins can inhibit sensitization to an oral allergen and can prevent the development of food allergies, while this protective effect was explained by an increase in the percentage of γδ TCR T cells, the main subset of intraepithelial T lymphocytes, which plays a crucial role in development of immune tolerance [104,130,225].

Considering that impairment of the intestinal barrier and increase in intestinal permeability have been known as the primary risk factors contributing to food allergy, it can be assumed that dietary polyphenols due to their ability to enhancement the intestinal barrier integrity and function might prevent the development or attenuate the symptoms of food allergy by inhibiting allergen permeation. Indeed, evidence from in vitro and animal studies has indicated that dietary polyphenols can alleviate intestinal barrier dysfunction and reduce intestinal permeability through different mechanisms including the upregulation of the intestinal tight junction (TJs) protein expression, increase in trans epithelial electrical resistance (TEER) across a cellular monolayer, reduction in oxidative stress and inhibition of signaling pathways such as nuclear factor kappa β (NF-κβ) and mitogen-activated protein kinases (MAPK) involved in the inflammation process [182,228,229,230]. The main evidence on the beneficial effect on intestinal barrier function and integrity and thus alleviation of food allergy symptoms are available for quercetin, luteolin, naringenin, kaempferol, curcumin, green and black tea flavonoids, grape seed proanthocyanidin, wild blubbery anthocyanins, and chlorogenic acids, tested in doses ranging from physiological (i.e., epicatechin) to pharmacological concentrations (i.e., berberine) [188,189,225,230,231,232,233,234]. Recently, the capacity of polyphenols to prevent food allergy by regulating intestinal immunity and improving intestinal barrier function has been well illustrated by studies carried out with olive oil, one of the main components of the Mediterranean diet, containing a high concentration of polyphenols such as phenolic acids (ferulic and caffeic), lignans and flavones (apigenin, luteolin) [68]. Two animal studies have demonstrated that olive oil supplementation decreased the scores of food allergy symptoms such as scratching and gastrointestinal response as well as improved the intestinal barrier integrity by repairing ileum tissue villi, upregulation of TJ protein expression, and decreasing mucin production. Moreover, olive oil reduced the Th2-cytokine level in lamina propria and alleviated the degree of tissue inflammation, whereas it upregulated the Treg population and increased the intestinal sIgA production, thus promoting the development of antigen tolerance and the maintenance of intestinal immunity [226,235]. In addition, studies in weaning piglets have revealed that apple and red wine polyphenols added to the starter diet can impact gut barrier architecture and function as well as suppress the GALT activation in Peyer’s patches in the ileum, resulting in the prevention of intestinal inflammatory response and a faster development of immune tolerance [130,236].

Overall, there appears to be promising evidence suggesting that dietary polyphenols may be an effective strategy for preventing and/or treating food allergies (Table 2). However, most findings are based solely on experimental studies and should be confirmed by high-quality human clinical trials, which are still lacking. Such efforts are particularly important because dietary interventions aimed at both preventing the initial stage of an allergic reaction, such as sensitization to a food allergen, and effectively treating food allergy symptoms are likely to exhibit an additional effect later in life by reducing the risk of developing allergic rhinitis and asthma.

## 6. Polyphenols in the Prevention and Treatment of Respiratory Allergy

Considering the fundamental role of allergic inflammation in the development and progression of respiratory allergic diseases, it can be assumed that polyphenols, due to their anti-inflammatory and immunomodulatory properties, may be beneficial in the prevention and treatment of asthma and allergic rhinitis. In addition to anti-allergic actions, in vitro studies suggest that polyphenols also can function as mucus anti-secretory agents and have antioxidant and antifibrotic activities and thus target not only allergic inflammation, but also the accompanying inflammation-induced oxidative stress and structural changes in the airways, leading to airway hyperreactivity and airway remodeling [130].

### 6.1. Allergic Rhinitis

Among flavonoids, quercetin is the most frequently studied in relation to allergic rhinitis due to its well-known anti-inflammatory and antihistamine properties [106,237]. The therapeutic effect of quercetin has been demonstrated in an experimental rat model of allergic rhinitis, in which orally administered quercetin reduced the nasal symptoms such as sneezing, rubbing and redness as well as alleviated allergic reaction by decreasing IgE and Th2-cytokine production, inhibiting the inflammatory cells infiltration and improving the imbalance of Th1/Th2 and Treg/Th17 [135,164,238]. In another experiment on allergen-sensitized rats, quercetin has been suggested to attenuate the symptoms of allergic rhinitis through the inhibition of neuropeptide productions and suppression of nasal neurogenic inflammation [239]. Further in vitro and in vivo studies have investigated the exact mechanism of the antiallergic effect of quercetin by using human nasal epithelial cells (HNEpC) and mice models of allergic rhinitis. Based on the obtained results, three potential mechanisms for the inhibitory effect of quercetin on the development of nasal allergy-like symptoms were proposed, which include reducing the production of NO by nasal epithelial cells and increasing the ability of nasal epithelial cells to produce endogenous proteins such as thioredoxin (TRX) and uteroglobin (Clara cell protein 10), which are known to suppress inflammatory cell chemotaxis and downregulate Th2 cytokine responses [240,241,242]. Recently, a study evaluating the therapeutic effect of onion extract, a rich source of quercetin, in a mouse model of allergic rhinitis demonstrated that topical administration of onion extract on the nasal cavity is efficacious in the treatment of allergic rhinitis symptoms by decreasing the level of specific IgE and Th2 cytokines and reducing eosinophil infiltration of the nasal mucosa [243]. The antiallergic properties similar to onion, resulting from equal content of quercetin compounds, were also confirmed in the case of shallots, which additionally proved to be effective in a preliminary clinical study in patients with allergic rhinitis. An oral shallot supplement (3 g/day) used in combination with a standard dose of antihistamine (cetirizine) improved symptoms such as sneezing, rhinorrhea, itchy nose, and eyes significantly more effectively than placebo and cetirizine alone [244]. Previous clinical studies evaluating the efficacy of enzymatically modified isoquercitrin in patients with Japanese cedar pollinosis have indicated that isoquercetin exerts both therapeutic effects in improving nasal and ocular symptoms during the cedar peak season and also prevents the development of symptoms when treatment was started 3 weeks before the first day of pollen dispersal [245].

Furthermore, evidence from in vivo studies has revealed that other flavonoids, such as luteolin, myricetin, naringenin, baicalin, rosmarinic acid, procyanidins, and catechin, were also able to reduce nasal itching and sneezing frequency, infiltration of inflammatory cells, nasal mucosa thickness and mucus secretion as well as decrease the levels of allergen-specific IgE in murine models of allergic rhinitis [165,169,246,247,248,249,250,251,252]. These findings support the potential therapeutic application of flavonoids in the treatment of allergic rhinitis; however, the clinical studies are limited. Promising results come from a randomized clinical trial assessing the therapeutic potency of tomato extract rich in naringenin, which indicated that oral administration of the extract for 8 weeks markedly reduces sneezing, rhinorrhea, and nasal obstruction and improves quality of life in subjects with persistent symptoms of allergic rhinitis due to house dust mite allergy [253]. Another randomized clinical trial of silymarin, a mixture of three flavonoids derived from milk thistle (silibinin, silydianine, and silychristine), demonstrated its effectiveness in attenuating the severity of allergic rhinitis symptoms [254]. A preliminary clinical study has shown that Pycnogenol^®^, a standardized extract from French maritime pine bark containing a mixture of flavonoid compounds (mainly procyanidins and catechins), alleviates the symptoms of allergic rhinitis in patients allergic to birch pollen, and, importantly, the effectiveness of the extract was found to be greater if treatment was initiated at least 5 weeks before exposure to the allergen [255]. Lertal^®^, a novel oral nutraceutical containing quercetin, vitamin D3, and Perilla frutescens (a mixture of rosmarinic, luteolin, and apigenin), was found to be effective in the reduction in allergic rhinitis symptoms and the need to use symptomatic medications in children observed during Phase II of a randomized, double-blind, placebo-controlled study [256]. Polyphenols extracted from apples, which consist primarily of procyanidins, tannins, catechins, and epicatechins, are also suspected to be effective in the treatment of allergic rhinitis as they have been reported to inhibit mast cell activation and histamine release [257]. Two randomized clinical studies confirmed that high-dose (at least 200 mg/day) consumption of apple polyphenols significantly attenuates nasal symptoms including sneezing, rhinorrhea, and swelling of the nasal turbinates in both patients with persistent allergic rhinitis allergic to house dust mites and those with seasonal symptoms due to cedar pollen allergy [250].

Resveratrol is a non-flavonoid polyphenol considered to be a candidate for the treatment of allergic rhinitis, owing to its promising immunomodulatory function. Recent in vivo studies in murine models of allergic rhinitis have clearly demonstrated that orally administered resveratrol can reduce nasal symptoms, inhibit the secretion of proallergic mediators and cytokines, and reduce the number of inflammatory cells in nasal tissue samples [258,259]. These findings were confirmed in a randomized clinical trial conducted in children with allergic rhinitis caused by pollen allergy, in which intranasal treatment with resveratrol led to significant improvement in all nasal symptoms including itching, sneezing, rhinorrhoea, and nasal obstruction as well as reduction in the use of symptomatic medications [260]. Furthermore, a study in adults with allergic rhinitis supported the beneficial effect of resveratrol that not only alleviated the nasal symptoms, but also ameliorated the quality of patients’ lives [261].

Curcumin is another non-flavonoid polyphenol for which a favorable therapeutic effect on allergic rhinitis has been reported in studies on animal models, pointing out that treatment with curcumin resulted in suppression of allergen-induced allergic rhinitis symptoms and histopathological features such as goblet cell metaplasia, infiltration of the inflammatory cell and vascular proliferation in nasal tissue [150,172,262]. Wu et al. conducted a randomized, double-blind study in allergic rhinitis patients, providing evidence that orally administered curcumin is able to modulate immune response, remarkably mitigate the nasal symptoms (sneezing, itching, rhinorrhea), and increase the nasal airflow, thus relieving the obstruction [263]. Recently, the safety of topical application of curcumin on the nasal mucosa has been experimentally confirmed since curcumin applied at appropriate concentrations did not exert adverse effects on the viability and proliferation of normal cells [264]. Moreover, an experimental study in a mice model of allergic rhinitis has suggested that using curcumin along with an allergen in the combined formulation results in better immunomodulatory effects and enhances the effectiveness of immunotherapy [132]. Table 3 summarizes current research that has been reported with regard to allergic rhinitis and different polyphenols.

### 6.2. Asthma

With reference to asthma models, resveratrol has been widely investigated in preclinical studies and has been proven to exhibit therapeutic activity against asthma [265]. Numerous studies in a mouse model of asthma have demonstrated that oral administration of resveratrol during the OVA challenge markedly reduced symptoms of airway hyperresponsiveness by suppression of peribranchial inflammatory cells infiltration, reduction in mucus production, relaxation of the respiratory tract smooth muscle and alleviation of allergic inflammatory response [265,266,267]. Moreover, resveratrol effectively suppressed airway remodeling observed in the course of asthma through ameliorating many structural changes in the airways such as epithelial damage, thickening of epithelium and the subepithelial smooth muscle, goblet cell hyperplasia, and hypertrophy [265,267,268,269]. Recently, Zhang et al. confirmed the protective antioxidant effects of resveratrol in a house dust mite (HDM)-induced asthma model, indicating that treatment with resveratrol prevented oxidative DNA damage and apoptosis in bronchial epithelial cells exposed to HDM [270]. Despite promising findings from in vitro and animal studies, research in human subjects is still lacking.

A considerable amount of data about effectiveness in asthma, both from preclinical and clinical studies, have been provided for curcumin. In a mouse model of allergic asthma, curcumin exerted a beneficial therapeutic effect concerning symptoms, airway inflammation, oxidative stress, and lung pathological changes (inflammatory cell infiltration and mucus hypersecretion) as well as airway constriction and hyperreactivity, mainly through inhibiting Th2 signaling pathways and inducible nitric oxide synthase and Treg cells stimulation [158,173,271]. Importantly, evidence suggested that curcumin, by inhibition of the Notch1–GATA3 signaling pathway, is capable of not only attenuating the severity of airway inflammation, but also preventing the development of an allergic inflammatory response when administered before the OVA challenge [272]. Curcumin has also been reported to be efficacious in the reduction in airway remodeling in asthma, with the effect of curcumin on histopathological features being dose-dependent and, at the highest doses, comparable to that of dexamethasone [171,271,273]. Moreover, Wu et al. showed that supplementation of curcumin in asthmatic mice could increase the therapeutic efficacy of dexamethasone and even prevent adverse effects caused by glucocorticoid treatment, suggesting potential employment as an add-on therapy in asthma [171]. Indeed, a randomized control study involving patients with mild to moderate asthma demonstrated significant enhancement in the mean FEV1 values in patients treated with oral curcumin in addition to standard therapy compared to a group receiving only standard inhaled therapy for asthma. Importantly, any significant side effects related to curcumin were not reported during the study, indicating the great safety profile of high-dose curcumin supplementation (1000 mg/per day) [274]. Next, in a double-blinded, placebo-controlled, randomized trial conducted in children with persistent asthma, oral administration of encapsulated curcumin as add-on therapy to standard treatment of asthma effectively improved disease control after 3 and 6 months, which was evident by less frequent nighttime awakenings and less frequent use of short-acting β-adrenergic agonists when compared to placebo [275]. In addition, administration of nutraceutical dietary supplements containing curcumin with resveratrol, soy phospholipids, zinc, selenium, and vitamin D in children with moderate to severe asthma was found to mitigate allergic airway inflammation as expressed by decreased fractional exhaled nitric oxide level [276]. Supplementation with high doses of curcumin (1500 mg twice daily) for 12 weeks is currently being investigated in a phase 2 clinical trial to evaluate the effect on moderate to severe asthma in adults; however, no data are available for review to date [277].

Based on the findings from in vitro studies indicating the immunomodulatory activities of flavonoids, these polyphenolic compounds should also be considered as potentially effective preventive and therapeutic agents for asthma [251]. Indeed, several studies in OVA-induced asthmatic mice have shown that luteolin, hesperidin, glabridin, green tea catechins, and rosmarinic acid can significantly reduce both symptoms of bronchoconstriction and allergic airway inflammation level by decreasing the Th2 cytokine level, inflammatory cells infiltration, mucus secretion, interstitial fibrosis, and collagen deposition, accompanied by alleviation of airway hyperresponsiveness and lung function improvement [136,160,278,279,280,281,282,283,284]. Recently, in a house dust mite (HDM)-induced asthma model, epigallocatechin gallate (EGCG), the major flavonoid extracted from green tea, has been demonstrated to decrease specific IgE in the serum while increasing IL-10 levels in the BALF, upregulate the amount of Treg cells and expression of Foxp3 mRNA in the lung tissue, thus effectively ameliorating tissue injury, airway inflammation and airway hyperresponsiveness [285,286]. Further, in vitro and in vivo studies have indicated that dietary kaempferol, in addition to anti-inflammatory activities, can also exhibit inhibitory effects on oxidative injury of lung epithelium and seems to be effective in the reduction in fibrotic airway remodeling by suppressing bronchial wall and bronchial smooth muscle thickening, leukocytes infiltration, goblet cell hyperplasia and alveolar hemorrhage observed in the lung of OVA-challenged asthmatic mice [159,287,288,289]. Interestingly, evidence suggested that kaempferol and EGCG have the potential either to prevent the development of allergic airway inflammation when given orally 1 h before OVA sensitization or to treat OVA-induced asthma symptoms when administered during the challenge to previously sensitized mice [290,291]. Most in vivo studies carried out with quercetin have confirmed its beneficial effect on immunological aspects of asthma, including reduction in white blood cells and eosinophil recruitment into the BALF and lung tissue as well as regulation of the Th2/Th1 imbalance [102,127,140,292]. Quercetin was found to be dose-dependently effective in the inhibition of immediate and late-phase asthma responses, and this activity seems to be similar to that of cromolyn sodium and dexamethasone treatment [102,140]. The abovementioned pine bark Pycnogenol^®^ formulations have also been demonstrated as potential therapeutic nutraceutical agents for the treatment of asthma. Three randomized, placebo-controlled, double-blind trials involving asthmatic adults and children have revealed that treatment with Pycnogenol^®^ at a dose of 100 mg/day markedly improves the control of asthma symptoms and lung function and reduces the need to use rescue inhaler medication [251,293,294].

In addition to those mentioned above, other polyphenolic compounds that demonstrated a broad spectrum of anti-inflammatory and anti-allergic effects in vitro have recently been intensively investigated in animal and clinical studies. For example, dietary supplementation of BerriQi^®^ Boysenberry and apple juice concentrate, containing high amounts of anthocyanins, ellagitannins, and chlorogenic acids, in OVA-challenged asthmatic mice can suppress immune cell infiltration in the lung, tissue damage, and mucus production as a result of regulation of innate immune pathways [295]. Similarly, the chestnut inner shell extract, as a rich source of gallic acid (a phenolic acid) and ellagic acid (a tannin), was found to present the anti-asthmatic efficacy inhibiting the inflammatory response induced by OVA challenge and relieving asthma symptoms, such as airway hyperresponsiveness and mucus overproduction in the asthma mice model [296]. Therapeutic properties of magnolol, an active polyphenol extracted from *Magnolia officinalis*, which is traditionally used in Chinese medicine for the treatment of asthma and cough, were also confirmed in studies on OVA-induced asthmatic mice models that showed significant suppression of allergen-induced airway hyperactivity and inflammation along with reduction in lung tissue eosinophil infiltration, mucus overproduction, and collagen deposition [297,298]. More recently, Yu and Li presented the anti-asthmatic effects of punicalagin, a major polyphenol present in pomegranates, in an OVA-induced experimental model of asthma [299]. Subsequently, a randomized, double-blind, placebo-controlled trial was conducted in adults with persistent allergic asthma to assess the therapeutic activity of a pomegranate extract which is known to exhibit three times the anti-inflammatory and antioxidant activity of other polyphenol-rich food sources [300]. The findings have revealed that patients in the intervention group receiving supplementation with pomegranate extract (500 mg/day for 8 weeks) reported significantly fewer clinical symptoms, including daytime and nighttime shortness of breath as well as activity limitation due to asthma symptoms when compared to the control group [301].

Considering all the mounting evidence, it is possible to expect that polyphenols can constitute a promising adjuvant agent for treating or preventing respiratory allergic diseases (Table 4). However, additional studies are necessary to fully understand their preventive and therapeutic potential and evaluate their clinical effectiveness and safety.

## 7. Limitations and Future Challenges

In recent years, the effects of polyphenols, both as dietary components and supplements, on allergic diseases have extensively been investigated, providing a large amount of promising data. However, studying the biological impact of polyphenols, particularly in humans, presents certain limitations and poses several challenges that need to be addressed before translating the current knowledge into dietary or therapeutic recommendations.

The first, very difficult to avoid, limitation results from the fact that most polyphenols are supplied from dietary sources, mainly fruits, and vegetables, with very variable content of phenolic compounds which directly impact their distinct dietary intake [29]. This great variability and lack of knowledge about the precise polyphenol concentration in the food or their real intake creates a substantial challenge in terms of comparing the effects of different polyphenols found in food sources and offering exact recommendations about the most beneficial foodstuff. Therefore, further research is needed to establish and characterize natural sources of polyphenols, adequately standardize the polyphenolic extracts, and, most importantly, identify the active phenolic compounds and metabolites in the extracts responsible for the antiallergic effects.

Given the wide spectrum of biological actions exerted by polyphenols, further in-depth studies evaluating the mechanism of action, level of activity, and structure–activity relationship are needed to ensure the targeted and effective practical application of phenolic compounds. Furthermore, polyphenols need to be investigated in terms of routes of administration, target tissues, adequate doses, as well as the most appropriate composition of phenolic extracts, as it appears that a combination of polyphenols may lead to a more effective beneficial effect. The potential use of polyphenols as preventive and therapeutic interventions requires preclinical studies testing a wide range of doses to determine the maximum safe single dose and the long-term safety profile polyphenols, owning to their natural origin, are essentially considered to be non-toxic and safe, which is further supported by data from preclinical and clinical studies reporting good tolerability of the evaluated phenolic compounds with the advantages of no adverse effects and high safety [169,220,244,245,246,247,249,253,254,255,274,275,282,283,284,285,286,287,288,289,293,294]. However, the amount of data available in this area is limited, and additional research is needed to assess the overall toxicity, and content of toxic substances generated during the polyphenol extraction process or food processing.

With regard to the ability of polyphenols to reduce allergenicity through conjunction with allergic proteins, it should be mentioned that the binding modes between polyphenols and proteins present in food depend on the food processing methods and include both covalent and non-covalent interactions [71]. Generally, covalent allergen–polyphenol conjugates, formed under alkaline conditions or as an effect of enzymatic oxidation, should be preferred because they are irreversible and more stable when compared to non-covalent bonds that occur under acidic and neutral conditions [302]. In addition, polyphenol–protein interaction might be affected by external factors such as pH, temperature, ionic strength, and salt concentration; therefore, the most favorable conditions should be established to reduce allergenicity in food processing [74].

The bioavailability of polyphenols presents a major limitation challenging the studies on their effectiveness in both allergic animal models and human subjects, as the quantities of phenolic compounds present in the blood after ingestion are strongly influenced by several factors [29]. Firstly, the intestinal absorption of polyphenols depends on the type of dietary source and is affected by their low solubility in water; secondly, weak chemical stability along with rapid and extensive metabolism in the liver and intestinal epithelium restricts bioavailability [303]. Additionally, gut microbiota extensively metabolizes polyphenols into microbial derivatives of various polyphenols, further complicating their absorption and bioavailability and, consequently, their bioactivity, which may differ from the parent compounds [304]. However, available information on the activity of these metabolites is scarce so far, and more studies are needed to evaluate their potential activity and for a better understanding of the bidirectional relationship between polyphenols and microbiota. Further research should also focus on identifying a food source that ensures optimal absorption of natural plant polyphenols as well as investigating the strategies that can improve the bioavailability of phenolic compounds. Therefore, in recent years, several drug delivery systems, such as lipid-based carriers, polymer nanoparticles, and conjugate-based systems, are being investigated to enhance the bioavailability and efficacy of polyphenols with promising results [305,306,307].

Furthermore, the bioaccessibility, bioavailability, metabolism, and biological effects of polyphenols may be modified by interaction with other bioactive, even phenolic, compounds in the food matrix [308]. These interactions and the presence of other bioactive molecules in the diet must be taken into account when interpreting the results of experimental studies mainly focusing on the beneficial effect of a single phenolic compound. This caution also applies to the findings from clinical studies, as inter-individual variability in responses to phenolic compounds has been observed depending on the dietary pattern [29]. Moreover, it was reported that the response to polyphenols intake may vary between subject to subject as a result of individual microbiota composition and personal metabolic status dependent on variations in metabolic enzyme activity [309]. Thus, it is important to bear in mind these inter-individual discrepancies, as well as the potential influence of age and ethnicity, when planning future research. This approach is needed in order to both develop general recommendations regarding the consumption of polyphenols and also determine the possibility of their future use in very promising personalized nutrition or therapy.

However, since most studies are focusing on in vitro or murine models, there is still an urgent need for large, well-designed human clinical trials and population studies evaluating the clinical application potential of different polyphenolic compounds on food and respiratory allergies. These studies should focus on determining the efficacy and safety profile in various age groups and clearly establish optimum conditions and time windows for polyphenol intake that lead to the best prophylactic and therapeutic effects.

## 8. Conclusions

With advancing knowledge of the important role of diet and nutrition in the development and severity of allergic diseases, there is a growing amount of attention on the anti-allergic benefits of natural food components that can enhance the dietary and therapeutic management of allergic diseases. Among the dietary ingredients, polyphenols have come into the spotlight as the most extensive group of bioactive secondary metabolites with a broad spectrum of biological actions, including widely proven anti-inflammatory, antioxidant, and immunomodulatory properties. As reviewed, evidence from experimental and clinical studies reported in the literature so far confirms the great potential of polyphenols to be used either for preventive approaches (functional foods or supplements) or therapeutic interventions in relation to allergic diseases. Although currently available data offer exciting prospects for the future, further studies are needed to better understand their potential mechanisms of action, inter-individual differences in metabolism, and bioavailability to ensure widespread and effective use of polyphenols as pharmaceutical agents or dietary interventions. The future integration of polyphenol-rich foods into daily diets or in the formulation of functional foods and supplements seems to be very plausible, especially since progress in this area is driven by increasing public awareness about diet and the growing tendency to self-medicate with health supplements.

## Figures and Tables

**Table 1:** The list of main classes of polyphenols with anti-allergic properties and their dietary sources.

Polyphenols Classes	Type	Dietary Source	Reference
Flavonoids			
Flavonols	Quercetin Kaempferol Myricetin	apples, cherries, berries, apricots, cranberries, grapes, mango peel, onions, kale, tomatoes, broccoli, fennel, capers, okra, rocket, tea, red wine, beer, cocoa, bee pollen	[30,40,43,45]
Flavones	Luteolin Apigenin Baicalein	lemon, tangerine, and orange peel and pulp parsley, green pepper, celery, artichoke, lettuce watermelon, melon, cantaloupe, apples green and black tea	[39,45,46]
Isoflavones	Genistein Daidzein	legumes such as soybean green peas and black beans	[30,42,47]
Flavanones	Hesperidin Naringenin	citrus peel, tomato peels, seeds, spices aromatic plants: mint, chamomile	[38,48]
Flavanols	Catechin Epicatechin Gallocatechin Epigallocatechin	peels of grapes peels of apples grapes, seeds, roasted peanuts, almonds, pistachios green tea leaves chocolate, red wine, rosemary	[30,38,41,44]
Anthocyanidins	Cyanidin	grape skin, wine lees grape pomace blueberries, banana, strawberries, cherries, pears, cranberries, plums, beans, red cabbage	[38,39,49]
Non-flavonoids			
Phenolic acids	Gallic acid Ferulic acid Caffeic acid Curcumin	onions, black radishes, red fruits, citrus peels grapes (seed and skin) potato peel tea and fruit tea, coffee	[34,43]
Stilbenes	Resveratrol	grape by-products, red and white wine, berry fruits, strawberries	[44,50]
Lignans		grains and cereals: oat, wheat, rye, barley strawberries, apricots cabbage, broccoli, garlic olive	[38,50]

**Table 2:** Effect of polyphenols on food allergy (in vitro and in vivo studies).

Polyphenol	Polyphenol Dose	Study Type	Results/Observations	Side Effects	Ref.
Epigallocatechin, Epigallocatechin gallate	50 mg/day extracted from tea	In vivo BALB/c mice model of αs1-casein milk protein allergy	Significantly reduced levels of mast cell protease, histamine, specific IgE antibodies, and Th2 cytokines Reduced degree of pathological changes in the intestine	No data available	[218]
Ellagitannins Gallic acid	0.1%; 0.5%; 1.0% tea leaf extract: gallic acid 1659.0 mg/100 g dry weight ellagic acid 4622.7 mg/100 g dry weight	In vivo BALB/c mice model of egg allergy	Reduction in symptoms such as scratching, lethargy, and gastrointestinal signs Modulating the Th1/Th2 balance Increased percentage of the Treg subtype Enhancing intestinal IgA secretions	No data available	[133]
Curcumin	3 mg, 30 mg/kg *Curcuma longa* extract	In vivo BALB/c mice food allergy model	Reduction in food allergy symptoms such as decreased rectal temperature and anaphylactic response Inhibited IgE, reduced Th2-related cytokines, and enhanced Th1-related cytokine Maintaining Th1/Th2 balance	No data available	[219]
Resveratrol	2.5–40 μg/mL 5, 10, 20 mg *Abies georgei* extract	In vitro RBL-2H3 cells In vivo BALB/c mice	Reduced mast cell degranulation and release of β-hexosaminidase and histamine Suppression in the development of diarrhea upregulates the rectal temperature Decreased serum level of specific IgE, mouse mast cell protease-1, and histamine	No cytotoxic effect	[220]
Catechins	0.05% 0.1% areca nut extract via drinking water	In vivo BALB/c mice	Attenuated OVA-induced allergic responses, including diarrhea Reduced infiltration and degranulation of mast cells in the duodenum Suppressed specific IgE production and Th2 immune response	No data available	[222]
Flavonoids	100 g/kg cocoa beans powder	In vivo Brown Norway rats	Suppressed synthesis of specific IgE Suppressed Th2-related cytokines released from mesenteric lymph node and spleen cells	No data available	[223]
Baicalin	50, 100, 200 μ mol/L 20 mg/kg *Scutellaria baicalensis* extract	In vitro Caco-2 cells In vivo BALB/c mice food allergy model	Reduction in food allergy symptoms, serum IgE, and effector Th2 cells Up-regulation of Treg Enhancement of intestinal barrier function through the regulation of tight junctions	No data available	[224]
Anthocyanidins	1 and 5 mg/mL wild blueberry extract	In vitro Caco-2 cells	Enhancement of intestinal barrier function and integrity of the intestinal cell monolayer Reduced intestinal permeability, increased TEER, upregulation of claudin-1	No data available	[232]
Theaflavins	0.02–0.20% black tea theaflavin mixture via food powder	In vivo BALB/c mice	Reduction in food allergy symptoms: the severity of diarrhea Alleviating oxidative stress	No data available	[233]
Chlorogenic acid	50, 200 mg/kg pure isolated polyphenol	In vivo BALB/c mice model of shrimp allergy	Reduction in food allergy symptoms Decreased IgE level Regulation of AMPK/ACC/CPT-1 signaling pathway	No data available	[234]
Ferulic acid Caffeic acid Apigenin Luteolin	1–3 g/kg/day olive oil	In vivo BALB/c mice	Repaired ileum villi, and upregulated tight junction protein expression Increased levels of Treg-related cytokines (IL-10) in lamina propria Decreased levels of Th2 cell-associated cytokines in lamina propria Reduced Burkholderiaceae and increased Clostridiaceae in the intestinal microflora	No data available	[226]
	600 mg/kg/day olive oil	In vivo BALB/c mice	Reduction in food allergy symptoms decreased the IgE level, increased expression of intestinal tight junction proteins (Claudin-1, Occludin), increased levels of mucin 2 and β-defensin	No data available	[235]

**Table 3:** Effect of polyphenols on allergic rhinitis (in vitro and in vivo studies).

Polyphenol	Polyphenols Dose	Study Type	Results/Observations	Side Effects	Ref.
Quercetin	1, 10 and 50 mg/kg, p.o. pure isolated polyphenol	In vivo BALB/c mice	Decreased sneezing, nasal rubbing, and nasal redness frequency Decreased level of NO, decreased IgE and Th2-cytokine production	No data available	[135]
	20, 35, or 50 mg/kg/day pure isolated polyphenol	In vivo BALB/c mice	Reduced rubbing and sneezing Reduced IgE, histamine in serum Decreased number of inflammation cells and goblet cells in tissues Inhibited Th1/Th2 imbalance and Treg/Th17 imbalance	No data available	[164]
	80 mg/kg pure isolated polyphenol	In vivo Sprague–Dawley rats	Decreased secretion, sneezing, and itching Decreased IgE and Th2-cytokine production Decreased eosinophil count in the mucosa of the nasal turbinate	No data available	[238]
	25 mg/kg pure isolated polyphenol	In vivo Sprague–Dawley rats	Inhibited nasal rubbing movements and sneezing	No data available	[239]
	20 mg/kg pure isolated polyphenol	In vitro HNEpC In vivo BALB/c mice	Inhibited nasal symptoms and increased TRX levels in nasal lavage fluids	No data available	[240]
	100.0 pM, 1.0 nM, 10.0 nM, 100.0 nM pure isolated polyphenol	In vitro HNEpC	Reduced NO production Downregulated Th2 cytokine responses	No data available	[241]
	20, 40 μL red onion extract	In vivo BALB/c mice	Reduced allergic rhinitis symptom Decreased levels of IL-4, IL-5, IL-10, IL-13 Reduced eosinophil infiltration of nasal turbinate	No data available	[243]
	3 g/day shallot oral supplement	Clinical study	Improved symptoms such as sneezing, rhinorrhea, itchy nose, and eyes	No side effects, well-tolerated	[244]
	100 mg/day pure isolated polyphenol	Clinical study	Improved nasal and ocular symptoms Prevention of the development of symptoms	No side effects	[245]
Luteolin	10, 30 mg/kg pure isolated polyphenol	In vitro PBMC In vivo BALB/c mice	Decreased allergic symptoms and serum HDM-specific IgE Inhibition of IL-4 production	No side effects	[247]
	10 mg/kg pure isolated polyphenol	In vivo BALB/c mice	Decreased nasal sneezing frequency, nasal mucosa thickness, and levels of specific-IgE and IL-17A, increased IL-10 and Foxp3 expression, suppressed Treg/Th 17 imbalance	No side effects	[169]
Myricetin	50, 100, 200 mg/kg pure isolated polyphenol	In vivo BALB/c mice	Protected against histamine challenge, decreased serum level of total and specific-IgE Inhibition of mast cell degranulation, regulation of Th1/Th2 balance	No data available	[165]
Naringenin	100 mg/kg pure isolated polyphenol	In vivo Sprague–Dawley rats	Decreased level of serum total IgE, IL4 and IL5 Reduced desquamation, erosion, and eosinophilic cell infiltration in nasal mucosa	No data available	[248]
	360 mg per day of tomato extract	Clinical study	Significantly decreased sneezing score, rhinorrhea, and nasal obstruction Improved patients’ quality of life	No side effects	[253]
Baicalin	100 μg/mL pure isolated polyphenol	In vitro PBMC In vivo BALB/c mice	Restored Th17/Treg cell balance Reduced infiltration of inflammatory cells of the nasal lavage fluid, improved nasal mucosal thickness and mucus secretion	No side effects mild laxative effect	[249]
Silymarin (silibinin, silydianine, silychristine)	140 mg 3 times daily mixture extracted from milk thistle *Silybum marianum*	Clinical study	Significant improvement in clinical symptom severity	No side effects	[254]
Pycnogenol (procyanidins, catechins)	50 mg French maritime pine bark extract	Clinical study	Reduced symptoms of allergic rhinitis in patients allergic to birch pollen	No side effects	[255]
Lertal (mixture of quercetin, rosmarinic, luteolin, apigenin)	150 mg quercetin, 80 mg *Perilla frutescens* extract	Clinical study	Reduction in allergic rhinitis symptoms and the need to use symptomatic medications	No data available	[256]
Procyanidins Catechin Epicatechin	50, 200, 500 mg/day apple polyphenols extract	Clinical study	Reduced nasal symptoms including sneezing, rhinorrhea, and swelling of the nasal turbinates	No data available	[250]
Resveratrol	5, 30, 50 mg/kg pure isolated polyphenol	In vivo BALB/c mice	Decreased levels of histamine, specific-IgE, IL-4, IL-5, IL-13, IL-17, and inflammatory cell numbers (leucocytes, eosinophils, lymphocytes, and neutrophils)	No data available	[258,259]
	100 μL *Polygonum* *cuspidatum* extract	Clinical study	Significant reduction in nasal symptoms: itching, sneezing, rhinorrhea, and obstruction as well as the need to use antihistamine	No data available	[260]
	100 μL *Polygonum* *cuspidatum* extract	Clinical study	Significant reduction in nasal symptoms decreased IgE, IL-4, and eosinophil levels in the blood, improved the patient’s quality of life	No data available	[261]
Curcumin	500 mg/d pure isolated polyphenol	Clinical study	Significant reduction in nasal symptoms (sneezing, itching, rhinorrhea), and increase the nasal airflow, suppression of IL-4, IL-8, IL-10	No side effects	[263]

**Table 4:** Effect of polyphenols on asthma (in vitro and in vivo studies).

Polyphenol	Polyphenols Dose	Study Type	Results/Observations	Side Effects	Ref.
Resveratrol	30 mg/day pure isolated polyphenol	In vivo BALB/c mice	Inhibited OVA-induced airway inflammation and mucus production	No data available	[266]
	10, 50 mg/kg/day pure isolated polyphenol	In vivo BALB/c mice	Reduction in inflammation, inhibition of respiratory tract remodelling progression Reduced collagen production Decreased IL-4, IL-5, IL-13,TGF-β1 and eosinophil level	No data available	[268]
	100 mg/kg pure isolated polyphenol	In vivo C57/Bl16 mice	Reduced inflammation and eosinophil infiltration	No data available	[269]
	100 mg/kg pure isolated polyphenol	In vivo C57BL/6J mice	Prevention of oxidative DNA damage and apoptosis in bronchial epithelial cells exposed to HDM allergen	No data available	[270]
Curcumin	10, 20 mg/kg pure isolated polyphenol	In vivo BALB/c mice	Significant decrease in airway inflammation and oxidative stress Treg cell stimulation	No data available	[158]
	120 mg/kg pure isolated polyphenol	In vivo BALB/c mice	Reduced cytokine production (IL-4, IL-5, IL-13) Suppression in tissue eosinophilia and mucus hyperproduction	No data available	[173]
	200 mg/kg pure isolated polyphenol	In vivo BALB/c mice	Reduced total cell influx and number of lymphocytes, eosinophils, and neutrophils in BALF Reduction in airway inflammation	No data available	[272]
	800 mg pure isolated polyphenol	In vivo BALB/c mice	Alleviation of lung inflammation Significantly reduced number of eosinophils and the hyperproduction of goblet cells Decreased Th2-related cytokines IL-4, IL-5, and IL-13 and Th17 cytokine IL-17A production	No data available	[171]
	1000 mg/per day of pure isolated polyphenol	Clinical study adults	Significant improvement in the mean FEV1 values	No side effects	[274]
	30 mg/kg/day roots of *Curcuma longa*	Clinical study children	Improved disease control: less frequent nighttime awakenings, less frequent use of short-acting β-adrenergic agonists	No side effects	[275]
Luteolin	0.1 mg/kg pure isolated polyphenol	In vivo BALB/c mice	Significant decrease in IL-4, IL-5, and IL-13 in their lung homogenate and in inflammatory cell infiltration in lung tissue	No data available	[278]
	50, 100 mg/kg *Artemisia argyi* extract	In vivo BALB/c mice	Reduced inflammatory cell counts, Th2 cytokines, airway hyperresponsiveness and mucus hypersecretion	No data available	[279]
Glabridin	40 mg/kg *Glycyrrhiza glabra* (licorice) roots extract	In vivo Wistar rats	Decreased serum IgE levels and the expression of TNF-α, IL-4, IL-5 Decreased inflammatory cells in the blood and BALF	No cytotoxic effect	[292]
Epigallocatechin gallate	20 mg/kg green tea extract	In vivo BALB/c mice	Reduced asthmatic symptoms, lung inflammatory cell infiltration, level of inflammatory factors, and increased the percentage of Treg	No data available	[280]
	5, 50 mg/kg green tea	In vivo BALB/c mice	Decreased airway hyperresponsiveness, tissue injury, airway inflammation, eosinophil infiltrations Reduced specific IgE in the serum and BALF Upregulated amount of Treg cells and expression of Foxp3 mRNA in the lung tissue	No data available	[285,286]
Kaempferol	1–20 μM 10–20 mg/kg E. pungens leaf extract	In vitro BEAS-2B cells In vivo BALB/c mice	Improvement in symptoms of asthma Suppressed collagen deposition, epithelial excrescency, goblet hyperplasia, and fibrotic airway remodeling Decreased eosinophils and leukocyte numbers in blood and BLAF	No cytotoxic effect	[282,283,284,285,286,287,288,289]
	50, 250, 500 μg/mL 1, 10 mg/kg, p.o. *Crocus sativus* extract	In vitro PBMC In vivo BALB/c mice	Reduced nitric oxide level and inflammatory cytokines in the lung tissue Inhibited activation of NF-κB and STAT-1 in macrophages Reduced percentage of neutrophils and eosinophils in bronchoalveolar lavage fluid	No data available	[290]
Quercetin	25, 50 mg/kg pure isolated polyphenol	Neonatal asthmatic rats	Reduced total number of leukocytes, eosinophils, level of TNF-α, IL-6, nitric oxide synthesis and apoptosis, regulation of the Th2/Th1 imbalance	No data available	[292]
Pycnogenol (procyanidins, catechins)	100 mg/day French maritime pine bark extract	Clinical study adults	Improved disease control: less frequent nighttime awakenings, decreased number of days with PEF < 80% and days with asthma score > 1, less frequent use of salbutamol and additional asthma medication Improvement in the severity of chest symptoms, wheezing, dyspnea, and daytime symptoms	No side effects, well-tolerated	[293]
	1 mg/kg/day French maritime pine bark extract	Clinical study children	Decrease in symptom scores, increase in lung function FEV1, PEF	No side effects, well-tolerated	[294]
Anthocyanins, ellagitannins, chlorogenic acids	0.2 mg/kg human equivalent dose BerriQi^®^ Boysenberry and apple juice concentrate	In vivo BALB/c mice	Significantly decreased OVA-induced infiltrating eosinophils, neutrophils, and T cells in the lung, and mucous production	No data available	[295]
Gallic acid, ellagic acid	100, 300 mg/kg	In vivo BALB/c mice	Reduced inflammatory cytokines, IgE, and number of inflammatory cells Reduction in inflammatory cell migration and mucus secretion in lung tissue	No data available	[296]
Magnolol	12.5, 25, 50 mg/kg *Magnolia officinalis* extract	In vivo BALB/c mice	Reduction in allergic inflammation, decreased levels of Th2 and Th17 cytokines Suppression of allergen-induced airway hyperactivity, airway eosinophilic inflammation, airway collagen deposition, and airway mucus hypersecretion	No data available	[297,298]
Punicalagin	12.5, 25, 50 mg/kg pomegranate extract	In vivo BALB/c mice	Decreased inflammatory cell infiltration into BALF Reduced levels of Th2-derived cytokines and specific IgE levels Regulation of IL-4/STAT6 and Notch/GATA3 signalling pathways	No data available	[299]
Ellagic acid	500 mg/day pomegranate extract	Clinical study	Improved clinical symptoms of asthma like daily breath shortness, nocturnal breath shortness, and limitation of asthma-related activity Reduction in eosinophil, basophil, and neutrophil counts	No data available	[301]

## Data Availability

Not applicable.
